# An Ultra-Sensitive Modular Hybrid EMG–FMG Sensor with Floating Electrodes

**DOI:** 10.3390/s20174775

**Published:** 2020-08-24

**Authors:** Ang Ke, Jian Huang, Luyao Chen, Zhaolong Gao, Jiping He

**Affiliations:** 1 Key Laboratory of Ministry of Education for Image Processing and Intelligent Control, School of Artificial Intelligence and Automation, Huazhong University of Science and Technology, Wuhan 430074, China; keang@hust.edu.cn (A.K.); zhaolong_gao@hust.edu.cn (Z.G.); 2 School of Optical and Electronic Information, Huazhong University of Science and Technology, Wuhan 430074, China; chenluyao1991@hust.edu.cn; 3 Beijing Advanced Innovation Center for Intelligent Robots and Systems, Beijing Institute of Technology, Beijing 100081, China; jiping.he@bit.edu.cn

**Keywords:** EMG, FMG, hybrid sensor, floating electrodes

## Abstract

To improve the reliability and safety of myoelectric prosthetic control, many researchers tend to use multi-modal signals. The combination of electromyography (EMG) and forcemyography (FMG) has been proved to be a practical choice. However, an integrative and compact design of this hybrid sensor is lacking. This paper presents a novel modular EMG–FMG sensor; the sensing module has a novel design that consists of floating electrodes, which act as the sensing probe of both the EMG and FMG. This design improves the integration of the sensor. The whole system contains one data acquisition unit and eight identical sensor modules. Experiments were conducted to evaluate the performance of the sensor system. The results show that the EMG and FMG signals have good consistency under standard conditions; the FMG signal shows a better and more robust performance than the EMG. The average accuracy is 99.07% while using both the EMG and FMG signals for recognition of six hand gestures under standard conditions. Even with two layers of gauze isolated between the sensor and the skin, the average accuracy reaches 90.9% while using only the EMG signal; if we use both the EMG and FMG signals for classification, the average accuracy is 99.42%.

## 1. Introduction

There are still millions of amputees in the world. Currently, the only way to recover is through a prosthesis. The development history of artificial limbs spans thousands of years, gradually from a decorative to a functional prosthesis, and currently the intelligent prosthetic based on the concept of the human–machine interface (HMI) [1]. The idea of an intelligent prosthetic usually refers to the “intent control” of the prosthetic limbs, which means the user controls the prosthesis through the brain or healthy body movement intention rather than direct body motions [2]. Based on these high-level intent commands, there are a number of control methods and theories to accomplish the low-level control of rehabilitation and auxiliary robots [3,4], thus they work together to achieve full control of the prosthesis. In the past few decades, the primary technology of the HMI for prosthesis control has been surface electromyography (EMG). Portable multichannel EMG systems are widely used for prosthesis control from experimental research to clinical applications [5]. In an investigation of EMG-controlled upper-limb prosthesis, Farina et al. pointed out some important criteria needed for prostheses systems, such as intuitive, closed-loop, adaptive, robust real-time control (<200 ms), the minimum number of recording electrodes, limited complexity, and low power consumption [6]. The use of muscle electricity has a long history, and signal processing methods are also easy to obtain. However, because of the different physical conditions of these amputees, there still exist problems that cannot be solved by signal processing, such as the migration of electrical sensors, the skin impedance changes caused by sweating and contact, and the fatigue of muscles [7].

Forcemyography (FMG) monitors the radially directed muscle pressures produced by the deformation of muscle in cross-section that occurs during muscular contraction, which provides an alternative method to measure muscle activity. In a previous study, the FMG showed a high correlation (Pearson’s r > 0.9) with the EMG linear envelope during simultaneous recording of the same muscle [8]. According to many researchers, the FMG has been proved to be a safe, reliable, non-invasive interface for prosthetic control [9,10,11]. Different from the EMG, FMG is not affected by the changes of contact impedance due to sweat [9]. The most widespread monitoring method for FMG involves the force transducers that are based on resistive polymer-thick-film (RPTF) technology, e.g., force sensing resistor (FSR) from Interlink Electronics (FSR400 and FSR402) and Tecscan (Flexiforce sensor) [9]. The FSR has also been used in many other biomedical engineering applications that involve force measuring [12] due to its convenience. In addition to the RPTF, pneumatic pressure sensors [13], piezoelectric sensors [14,15], and optical fiber sensors [16,17] have also been reported for the FMG monitoring. FMG can be used for hand gesture recognition with a reduced number of sensors to obtain high accuracy. In [18], the author demonstrated the feasibility of a piezoresistive array armband for hand gesture recognition with three FSR-based sensors. Since both the EMG and FMG can be used for prosthesis control, there are also many studies on the comparison of the EMG and FMG; in some research, it has been shown that the FMG is more stable than EMG over time [19], while in other research, the EMG is more accurate for muscle fatigue modeling [20].

A single signal source often lacks reliability in a practical scenario, especially in the case of sensor failure; sensor fusion can provide redundant and complementary information to avoid uncertainty, thus increase the reliability [21]. Some previous researchers studied the feasibility of combining the EMG and FMG to improve the performance of hand gesture pattern recognition [22]. However, the signal acquisition system used for EMG–FMG data fusion is a discrete system, which means the EMG and FMG are two completely separate systems; this increased the size and complexity of sensor modules, even for FSR, because an extra mechanical fixation device and force probe are usually required, similar to the setup in [19]. It is necessary to develop integrated EMG and FMG hybrid sensors that can provide more integration; however, the research in this area is still lacking. In [23], the author proposed a novel co-located EMG–FMG sensing wearable armband; in this design, the FMG is recorded by a barometer cast in rubber, while the electrode of EMG is silver foil, and a flexible silver foil electrode was placed on top of the barometer’s rubber. This was the first prototype that recorded the FMG and EMG in the same muscle location. However, the FMG sensing modular is a commercial unit (TakkTile, USA); the signal acquisition circuit and sampling frequency of the FMG are defined by the manufacturer and the circuit is independent of the EMG, which decreases the integration of the circuit design. Furthermore, the single sensing unit in this design has only one electrode for EMG, which means at least three sensing units are needed when recording the EMG signal in differential input mode—this reduces the modularity.

This paper presents a modular, integrated EMG–FMG measurement system with floating electrodes; in other words, the electrodes are not fixed to the shell. The overall system consists of eight identical hybrid EMG–FMG sensor modules and a control board. The hybrid EMG–FMG sensor module functions in EMG and FMG signal conditioning and amplifying, while the control board is used for analog-to-digital conversion and wireless data transmission of EMG and FMG. To ensure the synchronicity of the EMG and FMG signals in time and space, we adopt an innovative floating electrode design for the mechanical structure of the sensor modules. The three electrodes are fixed to a beam, which can move up and down in a small range. The bottom of the beam is then fixed to an FSR, and a silicon pad is used for force transmission.

## 2. Materials and Methods

### 2.1. The Hybrid Sensor Design

#### 2.1.1. Circuit Design

**Figure 1:**
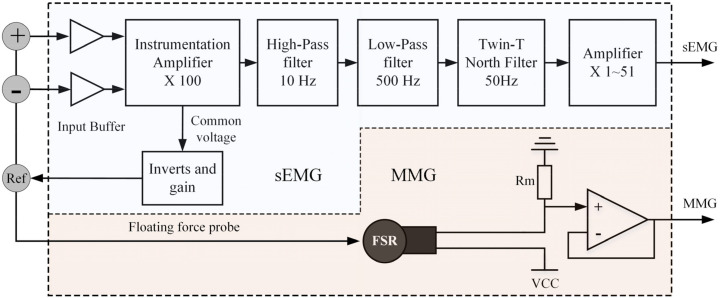
Block diagram of the hybrid FMG–EMG sensor module.

**Figure 2:**
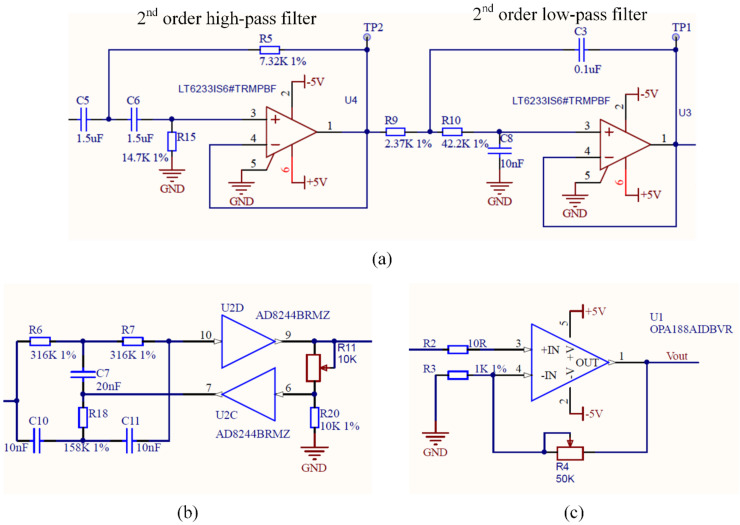
Part of the conditioning circuit of the EMGmeasurement circuit. (**a**) The band-pass filter. (**b**) The twin-T notch filter with adjustable quality factor (adapted from the datasheet of AD8244, in Figure 44, p. 17) [28]. (**c**) The second amplification stage with a changeable gain from 1 to 51; the gain value is set by R4.

**Figure 3:**
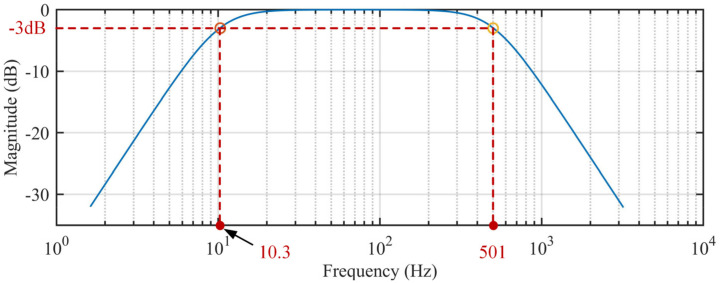
The frequency response of the proposed band-pass filter (10.3–501 Hz) simulation result.

Figure 1 shows the block diagram of the conditioning circuit for the hybrid EMG–FMG module. Since we use a dry electrode for EMG detection, the high contact resistance needs a large input impedance [24], so we connect an input buffer at each electrode (AD8244, ADI Company, Norwood, MA, USA) before the signal input to the instrumentation amplifier. The AD8244 is a precise quad unity-gain buffer that has ultra-large input impedance, and the input impedance is about 10 TΩ; the bias current of AD8244 is only 2 pA maximum. These characteristics make it suitable for EMG measurement. An instrument amplifier (AD8220, ADI Company, Norwood, MA, USA) with a gain of 100 is used to amplify the signal in the first stage. A 4th-order Butterworth band-pass filter (high-pass cutoff frequency at 10 Hz, low-pass cutoff frequency at 500 Hz) with unit gain designed with an accurate amplifier LT6233 (ADI Company, Norwood, MA, USA) is used to prevent hand movement artifacts and high-frequency noise; the circuit of the band-pass filter is shown in Figure 2a. The frequency response of the band-pass filter is shown in Figure 3. To remove the power line interference, we also adopt a twin-T notch filter after the band-pass filter; the circuit of the band-pass filter is shown in Figure 2b. The quality factor of the notch filter is adjustable; this circuit is adopted from the datasheet of AD8244. Finally, a variable gain circuit (OPA188) with a gain range from 1–51 is used; the gain is set by an adjustable resistor (0 to 50 KΩ), as shown in Figure 2c. The gain value of the second stage is set to about 26 during recording. The circuit of the reference electrode adopts the recommendation circuit in the AD8220 datasheet, in which the common-mode voltage at the midpoint of the AD8220 gain setting resistors is inverted by an operational amplifier (OP2177) and gained (OP2177), then driven back into the body; details on this circuit can be found in the datasheet of AD8220 (Figure 67, p. 25) [25]. This method is usually referred to as the right-leg drive circuit, which can help to cancel the common-mode signals.

Similar to many other studies, the FMG is measured by a commonly used FSR (FSR400, Interlink Electronics). The circuit used to extract the FMG signal is a voltage divider circuit recommended by the user guide due to its simplicity; some researchers suggested using a current-to-voltage recording circuit to reduce the hysteresis of FSR [9,26,27]. The force probe of FMG is acted upon by the floating EMG electrode at the same time.

#### 2.1.2. Mechanical Structure Design

**Figure 4:**
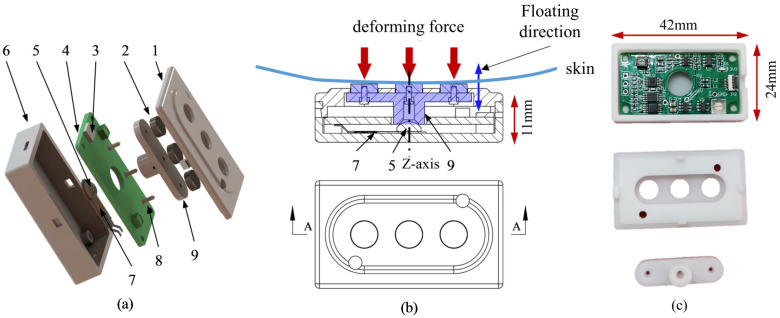
The mechanical structure of the hybrid EMG–FMG sensor. (**a**) The exploded view of the structure. 1: The cover structure, 2: The three electrodes, 3: The FPC connector, 4: The PCB, 5: The silicon pad that sticks on the center of the FSR, 6: The containing box, 7: The FSR, 8: The screw for fixing, 9: The force beam that transmits the force applied on the electrodes. (**b**) The illustration of the force transmission to FSR. (**c**) Photos of the fabricated shell (3D printed) and the PCB.

The central concept of the hybrid EMG–FMG sensing system is the floating electrode. Traditionally, for a dry electrode EMG recording system, the electrode is fixed to the mechanical structure, such as the DELSYS TRIGNO [29] and some other multi-modal EMG designs [30]. Here, we combine the function of the EMG electrode and the force probe; the three electrodes of the EMG system are fixed to a beam and can float along its z-axis in a very small range (about 0.5 mm), as shown in Figure 4. The force beam contacts a 3 M self-adhesive silicon rubber pad (5 mm in diameter and 2.3 mm in height, with a sphere dome). The rubber pad is attached to the FSR, which is then fixed at the bottom of the containing box; the PCB has a mechanical hole of 8mm diameter at its center to allow the beam to pass through. Since the rubber pad is elastic, it will act as a short “spring”, and the electrode will remain in place beneath the preload while there is no external force acting on it. The surface electrodes used for EMG signal recording are cylinder stainless steel electrodes from NeuroSky; the diameter of the electrode is 6 mm, and the height is 2 mm. The mechanical size of the sensor module is about 42 mm × 24 mm × 11 mm.

#### 2.1.3. Control Board

**Figure 5:**
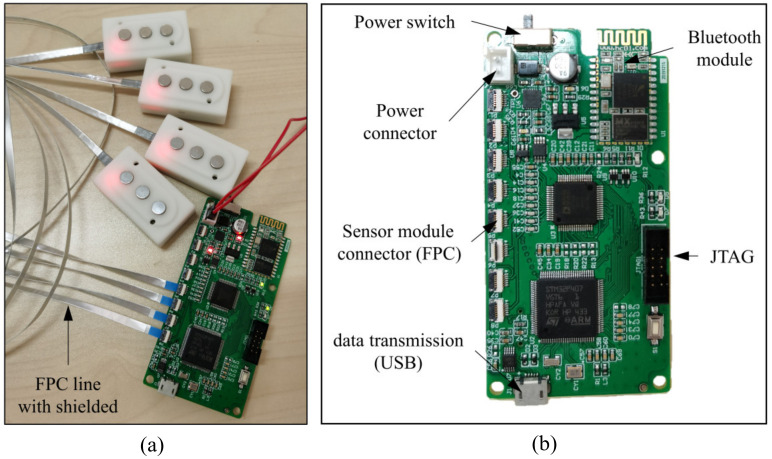
(**a**) The picture of the overall system with the control board and sensor modules. (**b**) The picture of the control board and the illustration of the connectors.

The main components of the control board are the microcontroller STM32F407 and the AD7606. The microcontroller (MCU) STM32F407VG from STMicroelectronics has a 32-bit Cortex-M4 CPU, and the working frequency reaches 168 MHz. An eight-channel with a 16-bit synchronous sampling analog-to-digital converter (AD7606) is used for converting the analog EMG and FMG signal to a digital signal. The AD7606 can work at a sampling frequency of 200 kSPS for all channels; in this work, the sampling frequency is set to 1 KHz. The wireless transmission interface we adopt here is the HC-06 Bluetooth module. The Bluetooth module exchanges data with the microcontroller through the serial port, which can operate at a baud rate of 921,600. A remote host desktop computer receives the data through another Bluetooth adapter. The general appearance of the control board and the sensor module is shown in Figure 5.

### 2.2. Experiment and Performance

#### 2.2.1. EMG vs. FMG Signal Comparison

**Figure 6:**
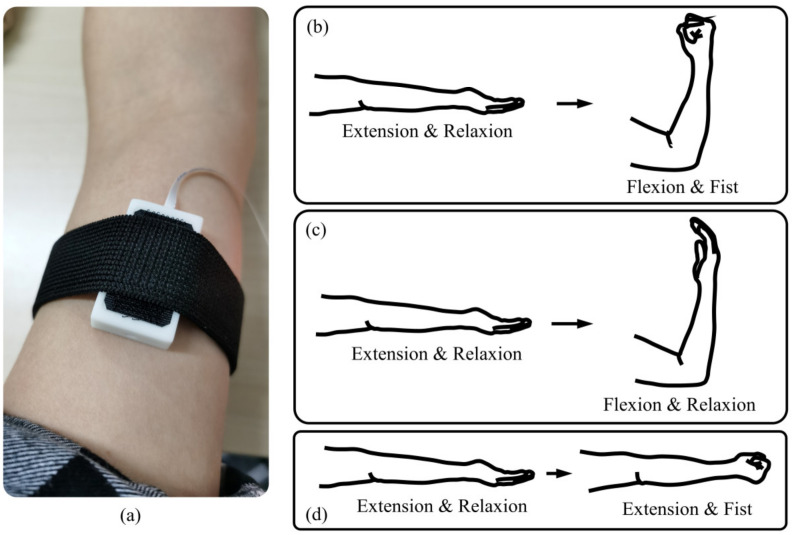
Explanation of the signal comparison experiment. (**a**) One sensor unit was attached to the biceps brachii. (**b**) Situation 1, elbow flexion and extension with the hand in a fist and relaxed. (**c**) Elbow flexion and extension with the hand relaxed. (**d**) Hand relaxes and is then made into a fist.

In order to provide an intuitive comparison of the EMG signal and FMG signal, we conducted a simple experiment to show the characteristics of EMG and FMG under different conditions. The subject was required to perform elbow flexion/extension, and hand in a fist/relaxed with different combinations. The three movements conducted for comparison are described below:

1. Elbow flexion and extension with the hand in a fist and relaxed. As illustrated in Figure 6b, the subject keeps the hand in a tight fist during elbow flexion but relaxes it while performing elbow extension.
2. Elbow flexion and extension with the hand relaxed. As illustrated in Figure 6c, the subject keeps the hand relaxed during elbow flexion and extension.
3. Hand relaxes and is then placed in a fist. As illustrated in Figure 6d, the subject keeps the elbow extension fully in a natural position, then places the hand in a fist and relaxes periodically.

**Figure 7:**
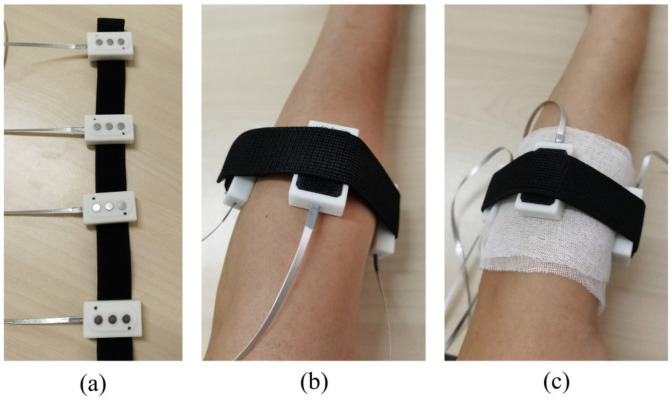
The explanation of two experimental conditions for hand gesture recognition (**a**) The four sensors were attached to an elastic belt with hook and loop fasteners. (**b**) Condition 1: the electrodes of the sensors directly contacted the skin. (**c**) Condition 2: the skin was wrapped in two layers of medical gauze; the electrodes of the sensors contacted the skin indirectly.

For all three conditions, the subject keeps the arm lying on the desktop at a suitable height. The contact area of the biceps brachii was first wiped with scrub cream to remove the stratum corneum and then cleaned with alcohol. One sensor module was fixed on the biceps brachii with an elastic cloth to measure the EMG and FMG signals, as shown in Figure 7a. During the elbow flexion and extension, there was no load applied to the subjects.

#### 2.2.2. Gesture Recognition

**Figure 8:**
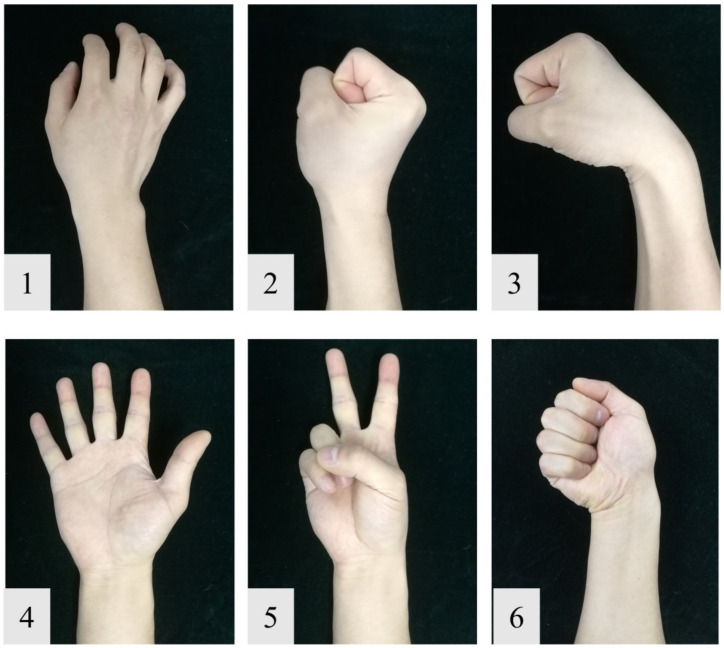
The gestures used in our experiment. (**1**) Relaxing. (**2**) Fist. (**3**) Wrist flexion. (**4**) Hand open. (**5**) Two fingers. (**6**) Ulnar deviation.

The experiment was conducted under two conditions: one condition is that the skin was well prepared. The skin was first cleaned by skin preparation gel (NuPrep, Weaver and Company, Aurora, CO, USA) and then wiped by alcohol, as shown in Figure 7b; the other is that the skin was wrapped in two layers of medical gauze; this simulates the scenario with clothing isolated, as shown in Figure 7c. The subjects were required to perform a series of six gestures following video cues on a computer (including the relaxing gesture) shown in Figure 8. The hold time of each gesture was three seconds, followed by three seconds relaxing; each gesture was performed ten times. After each trial, there was a brief relaxing time of about two minutes for subjects to avoid muscle fatigue. In this study, only four sensors were actually used for data acquisition. The sensors were not placed onto specific muscles, but were rather evenly distributed around the forearm. The sensors were attached to an elastic cord with hook and loop fasteners.

#### 2.2.3. Data Acquisition

Eight healthy right-handed volunteers without upper arm motor dysfunction history (five males, three females) were recruited for our experiment, all subjects were informed about the experimental procedures and associated risks before anticipating. After the preparation of the experimental setup, the data recorded by the control board were transferred to a desktop computer using a Bluetooth connection; data were saved on the hard disk for offline evaluation. The data analysis was performed offline in Matlab (MathWorks Inc, Natick, MA, USA) on a desktop computer.

#### 2.2.4. Data Analysis

##### Comparison of EMG and FMG Envelopes

The comparison of the EMG and FMG mainly focused on the low-frequency component; the envelope of the EMG signal used for comparison with the FMG was obtained using the following steps: First, the direct current (DC) bias was removed (the best straight-fit line of the raw EMG signal; this procedure was achieved by the function detrend in Matlab) from the raw signal. Then the signal was rectified to positive by applying an absolute operation. Finally, the rectified signal was filtered through a low-pass filter (6-order Butterworth, with stop frequency of 5 Hz) to obtain the envelope; this procedure was performed by zero-phase filtering to avoid phase delay. Since the EMG and FMG signals are different physically, we just compared the normalized signal to find the correlation. The raw FMG signal and the envelope of the EMG signal were both smoothed with a moving average filter of 10 points (which means a moving average filter with a 10 ms span was used to smooth all the data; this procedure was achieved by the function smooth in Matlab) before normalization to the maximum value separately.

##### Feature Extraction

A sliding window method was used to calculate the features of the EMG and FMG signals; in this way, the raw EMG and FMG signals without any pre-processing were split with a pre-defined window length and step time. The window length and the step size are the most critical parameters for this method. The window length directly influences the accuracy while the step size determines the control delays. According to [31], controller delays shorter than 200 ms can be regarded as real-time control. As recommended in [32], the optimal window length was between 150 and 250 ms. Considering the acceptable controller delays, the time windows we used here were set to 200 ms, with a moving step size of 100 ms. Since the reaction time from changing of the video cues to the subject starting the corresponding movement was different for each subject, the motion switch duration of about 1 s (0.5 s before and after the gesture switch time in the video cues) was removed from the samples used for classifying.

The feature extraction of the EMG and FMG signals has been well researched and reviewed in many papers [9]. Generally speaking, the feature extraction method used for EMG can be divided into three types: time-domain (TD), frequency-domain (FD), and time-frequency domain (TFD). In most cases, TD features are simple and effective, and the TD feature required less computation, which makes it practical to be embedded in micro-controllers. In this work, we used the feature combination of the mean absolute value (MAV) and root mean square (RMS) for both EMG and FMG signals.

##### Classifier

Four commonly used classifiers were selected to evaluate the performance of our hybrid sensor. The classifiers we used include the most widely used linear Support Vector Machine (linear SVM), the cubic Support Vector Machine (cubic SVM), the k-Nearest Neighbor (kNN, k = 5), and Linear Discriminant Analysis (LDA). The total dataset was randomly partitioned into five equal-sized subsets. Then, we used four subsets as the training set and the last one for testing. This procedure was repeated one hundred times to calculate the average accuracy of each subject.

## 3. Results

### 3.1. Comparison of EMG and FMG Envelopes

**Figure 9:**
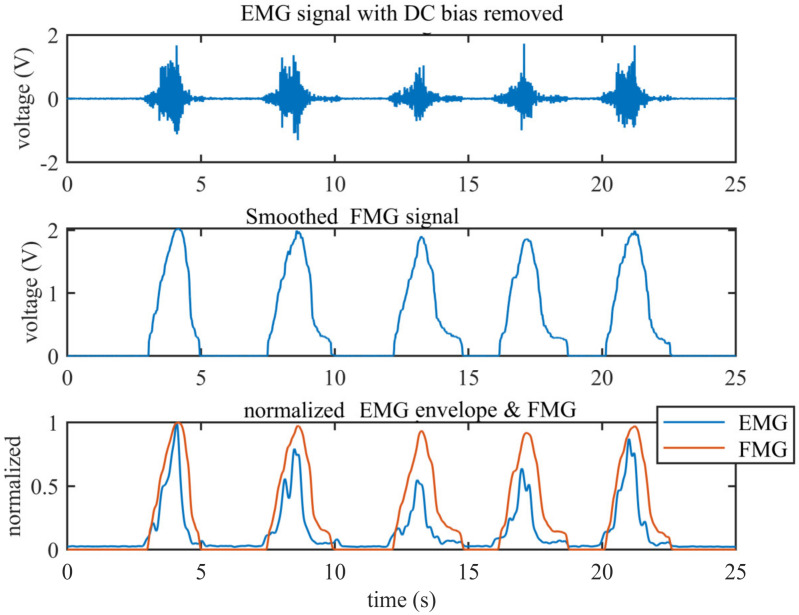
The raw EMG signal with DC bias removed and the raw FMG signal; the bottom subgraph shows the normalized EMG envelope and FMG signal.

Figure 9 shows the comparison of the EMG and FMG signals measured as the biceps brachii during elbow flexion and extension. The top subgraph shows the raw EMG signal measured by the sensor (the DC bias of the signal has been removed). It can be seen from the graph that the baseline of EMG is very steady and small. The middle subgraph shows the synchronous FMG signal, which shows a high correlation with the EMG signal. The bottom subgraph shows the comparison between the normalized FMG signal and the normalized envelope of the EMG signal; the correlation coefficients of this sample segment of the normalized FMG and EMG envelopes is 0.8763.

### 3.2. Comparison of EMG and FMG Signals under Different Muscle Loads

**Figure 10:**
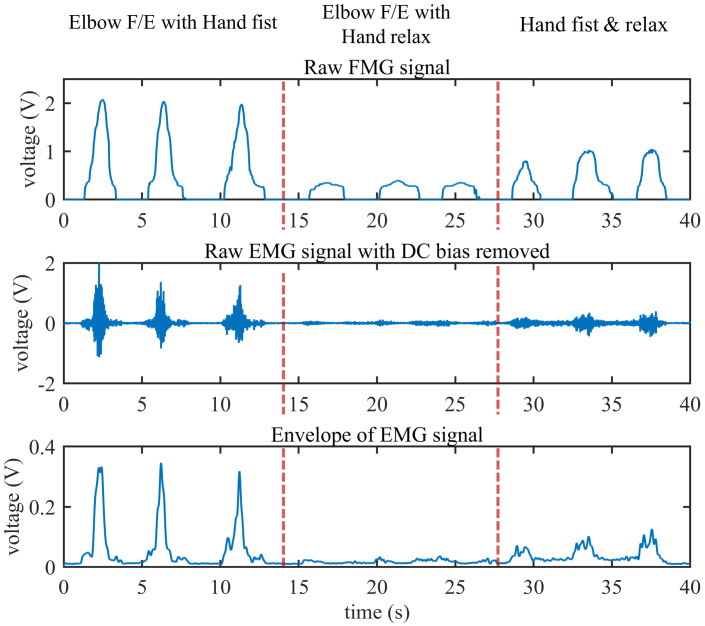
The comparison of the EMG and FMG signals under three different movements.

Figure 10 shows the comparison of the EMG (with DC bias removed) and raw FMG signals in three different movements. In this experiment, the muscle is under isotonic contraction; if the hand is in a tight fist, the muscle force at the biceps brachii will be larger than the condition when the hand relaxes. The level of muscle force is also reflected in the EMG and FMG signals. It can be seen from Figure 10 that the struggle force of the muscle affects both the FMG and the EMG signal. However, the average amplitudes of the FMG signal under these three conditions (corresponding to movement 1 to 3 in Section 2.2.1.) are 2.030 V, 0.364 V, and 0.961 V, respectively, while the average amplitudes of the EMG envelope are 0.321 V, 0.020 V, and 0.088 V, respectively. The ratio of elbow flexion and extension with the hand in a fist to the hand relaxed is 5.6 for FMG and 16.05 for EMG. It seems that the struggle force has less influence on FMG than on EMG. From another point, both the EMG and the FMG signal contains information about the muscle strength.

### 3.3. EMG Signal under Limiting Conditions

**Figure 11:**
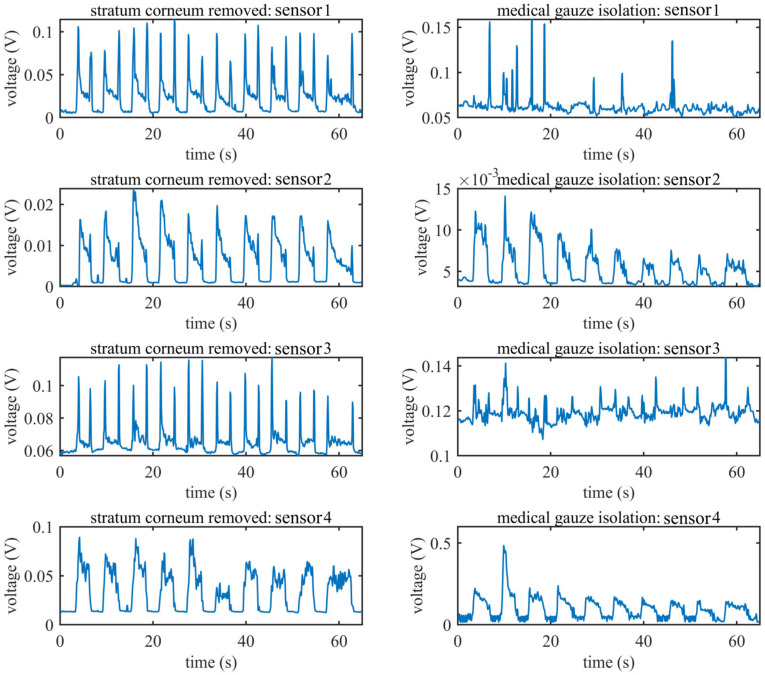
The RMS features computed from the EMG signal of all sensors of subject F when performing ulnar deviation; the left column shows the signals recorded under standard conditions; the right column shows the signals recorded with medical gauze isolated between the sensors and skin.

Figure 11 shows the RMS features of the EMG signal condition when subject F conducted ulnar deviation under two conditions. The four subfigures in the left column show the signals recorded under the standard condition while the four subfigures in the right column show the signal recorded with medical gauze isolation. Compared with the standard condition, the signals recorded at sensors 1 and 3 with medical gauze isolation are very poor, but there are still some sensors that contain information about hand movements, such as sensor 2 and sensor 4; the amplitude of the RMS feature under the medical gauze isolation condition is even higher than that under the standard condition for sensor 4. We suspect the reason for this is that the skin of subject F was slightly wet due to sweat during the experiment because we found the gauze was a little damp after the experiment.

### 3.4. Gesture Recognition Performance

Table 1 shows the average classification accuracy (mean and standard deviation) for all subjects; the features of both EMG and FMG are TD features (MAV+RMS). It can be seen from the table that the highest mean accuracy of our hybrid sensor when using both the EMG and FMG signal is more than 97% for all classifiers. It is impressive that the performance of the hybrid sensor is almost unaffected by the gauze. The average accuracy only decreases by about 5%. The cubic SVM has the best performance among the four classifiers; the best average accuracy of the hybrid sensor source is higher than 99% when using the cubic SVM for classifying. For all classifiers and measurement situations, the hybrid signal performs better than a single source (except for one condition: gauze isolation with the kNN classifier). It is also impressive that even with two layers of medical gauze isolated between the sensors and the skin, the classifier accuracy of EMG can reach 90.9%. This is consistent with the previous section. The EMG sensor can detect weak signals under some extreme conditions.

Table 2 shows the average classification accuracy (mean and standard deviation) for each subject when using the cubic SVM for classifying. For all subjects, the accuracy of the hybrid signal is better than 98%. The accuracy of the hybrid source signal is better than every single source. It can also be seen from the table that the FMG has a more robust performance than EMG under two conditions for almost all subjects. To our surprise, under the condition that the sensor is isolated from skin by gauze, for subject E and subject F, the accuracy with EMG is 98.15% and 97.97%, respectively, and even the lowest accuracy is about 84.27% (subject G).

## 4. Discussion

According to the principle of EMG, there will be no useful signal if the electrodes are insulated from the skin, and the surface EMG signal will also be significantly influenced by sweat or the stratum corneum. However, our experiments showed that when using a very high impedance buffer as the input stage of the analog front-end, the sensor could also record useful signals for gesture recognition.

For research on intelligent prosthetic control, the goal is to provide a robust, safe, accurate, and fast response HMI scheme. Usually, many studies focused on a highly effective algorithm; however, it is also essential to focus on the sensor itself; a high-performance sensor will depend less on the algorithm and hardware. The control system of an intelligent prosthesis is often an embedded system; the size, weight, and power consumption requirements mean the algorithm should not to be very complex and challenging to be embedded in a microcontroller. Based on this, our design provides an excellent choice for prosthetic control, because high accuracy can be achieved by using simple TD features and SVM classifiers.

Although we have demonstrated the performance of the sensor, there are still many aspects that need to be accessed. In real-life scenarios, the movement of the users, the sweating of skin, the displacement of the electrode, and muscle fatigue all will have a severe influence on the signal of both EMG and FMG; whether the sensor can still perform so well under these conditions remains to be tested. Also, the data fusion method of EMG and FMG used in this work is simply a combination of the feature sets, and there may be other better data fusion algorithms to further improve the accuracy of gesture recognition.

## 5. Conclusions

This paper provided a novel and high-performance hybrid EMG–FMG sensor. Different from the previous designs in other research, we adopted an innovative design that allows the electrode of EMG to float, which then can be used for the force probe of FMG simultaneously. With this design, we could detect the FMG and EMG at the same position of the muscle.

Adequate experiments were conducted to evaluate the performance of the designed sensor. The signal correlation of the EMG and FMG was high (r = 0.8763); this confirmed the FMG can provide information comparable to that provided by EMG. The experimental results also showed that when the muscle force is small, the FMG signal could be more prominent than the EMG signal. To access the gesture recognition accuracy with our proposed sensor, we conducted a standard experiment that involved six gestures among eight subjects; the gesture recognition experiment was also conducted under the condition that the electrode and skin are separated by two layers of gauze. The experimental results showed that when using the simple feature set (MAV + RMS) and commonly used classifier (cubic SVM), the average accuracy is higher than 99% when combining the EMG and FMG signals under both standard and extreme conditions.

Furthermore, the results also indicated that the combination of EMG and FMG performed better than every single signal source, which provided evidence that a hybrid HMI sensor system will perform better than a single source. More impressive, even isolated by two layers of gauze, the sensor could also detect weak EMG signals. Although the signal did not compare that well to the signal under the standard condition, it still performed well in terms of gesture recognition; the average accuracy when using the EMG signal was about 90.9% under this condition.

## Figures and Tables

**Table 1:** The average classification accuracy (mean and standard deviation) for all subjects and all six hand gestures with different classifiers.

Classifier		LDA		kNN		Linear SVM		Cubic SVM	
Accuracy		Mean	Std	Mean	Std	Mean	Std	Mean	std
normal	EMG	0.8701	0.0708	0.9218	0.0515	0.9583	0.0350	0.9551	0.0301
	FMG	0.9420	0.0490	0.9667	0.0192	0.9601	0.0286	0.9739	0.0187
	EMG + FMG	0.9783	0.0162	0.9811	0.0124	0.9891	0.0071	0.9907	0.0050
gauze isolation	EMG	0.7187	0.1834	0.8201	0.0956	0.7856	0.1720	0.9090	0.0513
	FMG	0.9523	0.0365	0.9821	0.0113	0.9681	0.0188	0.9876	0.0075
	EMG + FMG	0.9763	0.0253	0.9757	0.0122	0.9850	0.0150	0.9942	0.0041

**Table 2:** The average classification accuracy (mean and standard deviation) for each subject for six hand gestures; the classifier is the cubic SVM.

Conditions		Skin Prepared			Gauze Isolation		
Features		EMG	FMG	EMG + FMG	EMG	FMG	EMG + FMG
Subject A	mean	0.9572	0.9915	0.9872	0.9010	0.9937	0.9973
	std	0.0140	0.0052	0.0072	0.0206	0.0075	0.0037
Subject B	mean	0.9399	0.9850	0.9926	0.8728	0.9955	0.9987
	std	0.0141	0.0069	0.0060	0.0198	0.0047	0.0024
Subject C	mean	0.9573	0.9423	0.9959	0.8427	0.9902	0.9915
	std	0.0127	0.0331	0.0046	0.0307	0.0084	0.0073
Subject D	mean	0.9912	0.9863	0.9948	0.9343	0.9803	0.9946
	std	0.0047	0.0087	0.0052	0.0173	0.0092	0.0058
Subject E	mean	0.9811	0.9689	0.9938	0.9815	0.9911	0.9973
	std	0.0089	0.0131	0.0061	0.0076	0.0072	0.0031
Subject F	mean	0.8919	0.9872	0.9861	0.9797	0.9754	0.9895
	std	0.0306	0.0081	0.0080	0.0092	0.0141	0.0071
Subject G	mean	0.9657	0.9808	0.9934	0.8724	0.9809	0.9879
	std	0.0146	0.0106	0.0050	0.0253	0.0092	0.0078
Subject H	mean	0.9568	0.9491	0.9821	0.8880	0.9934	0.9967
	std	0.0107	0.0144	0.0079	0.0195	0.0057	0.0042
